# Locoregional tumor burden and risk of mortality in metastatic breast cancer

**DOI:** 10.1038/s41698-022-00265-9

**Published:** 2022-04-05

**Authors:** Sherry X. Yang, Stephen M. Hewitt, John Yu

**Affiliations:** 1 Division of Cancer Treatment and Diagnosis, National Cancer Institute, National Institutes of Health, Bethesda, MD USA; 2 Laboratory of Pathology, Center for Cancer Research, National Cancer Institute, National Institutes of Health, Bethesda, MD USA; 3 ELIASSEN Group, Reston, VA USA

**Keywords:** Breast cancer, Prognostic markers

## Abstract

The role of lymph node involvement and tumor size in metastatic disease including breast cancer is unclear. Here, nodal metastasis and T stage on the risk of mortality were investigated in de novo metastatic breast cancer population (35812 patients) in the Surveillance, Epidemiology, and End Results (SEER) Program database in the United States. We found an association between all-cause mortality and regional node involvement (adjusted hazard ratio [HR] = 1.45, 95% confidence interval [CI] 1.36–1.55, *p* < 0.0001) or T stage (HR = 1.20, 95% CI 1.14–1.25, *p* < 0.0001), independent of known clinicopathologic measurements. Number of positive nodes, and size and chest wall involvement of the breast tumors exhibited similar significance for breast cancer-specific mortality in the population (*p* < 0.0001 each), and all-cause mortality in hormone receptor (HR)-positive/HER2-negative (HR^+^/HER2^–^), HR^+^/HER2^+^, HR^–^/HER2^+^ and triple-negative metastatic breast cancer subtypes. Thus, nodal involvement and T stage are independent risk factors for mortality in the population of de novo metastatic breast cancer.

## Introduction

Female breast cancer has overtaken lung cancer as the most diagnosed cancer, with an estimated 2.3 million new cases (11.7%) among 36 cancers in 185 countries^1^. In 2022, about 287,850 new incidences that represent 30% of all new cancer cases and 43,250 deaths estimated to occur in women in the United States^2^. It is the most frequently diagnosed and second leading cause of cancer mortality in women. Approximately 6% breast cancer patients presented with stage IV disease at diagnosis, known as de novo metastatic breast cancer^3^. In addition, 10 to 52% patients who were initially diagnosed with local and regional stages of breast cancer eventually develop distant recurrence^4–7^. Despite significant advances in breast cancer diagnosis and treatment^8,9^, 5-year survival rate in patients with de novo metastatic breast cancer was 27% in patients diagnosed during 2009 through 2015^3,10,11^. Noticeably, about 10 to 20% of patients with de novo stage IV breast cancer could survive for over 10 years^12^. Recurrent metastatic breast cancer had similar survival rate to the de novo disease if metastasis-free survival (diagnosis of distant metastasis) was more than 24 months and patients with metastatic-free survival less than 24 months had much worse survival rate^13^. In addition, de novo unfavorable liver, lung and brain metastases were not associated with worse survival in breast cancer subtypes by a large population-based study^14^. All the data underscore an immense need to identify risk factors in addition to the distant metastasis per se to improve clinical outcome of patients with metastatic disease^15–17^.

The lymph node involvement and extent of disease, rather than sites of metastasis, were associated with the response to treatment and/or had trends for poor prognosis (with relatively small sample sizes) in an early and recent studies^17–19^. Overall, nodal involvement and tumor size in relation to the risk of mortality is undescribed systemically in metastatic breast cancer at diagnosis. We hypothesized that locoregional tumor burden was a risk factor for mortality and associated with negative outcomes in patients with metastatic breast cancer. We systemically evaluated the association of regional lymph node status, and size and chest wall involvement of the breast tumor (T stage) with the risk of all-cause mortality and breast cancer-specific mortality in de novo metastatic breast cancer population. Their association with the time-to-death events was also assessed in metastatic breast cancer subtypes.

## Results

### Characteristics of de novo metastatic breast cancer population

**Fig. 1:**
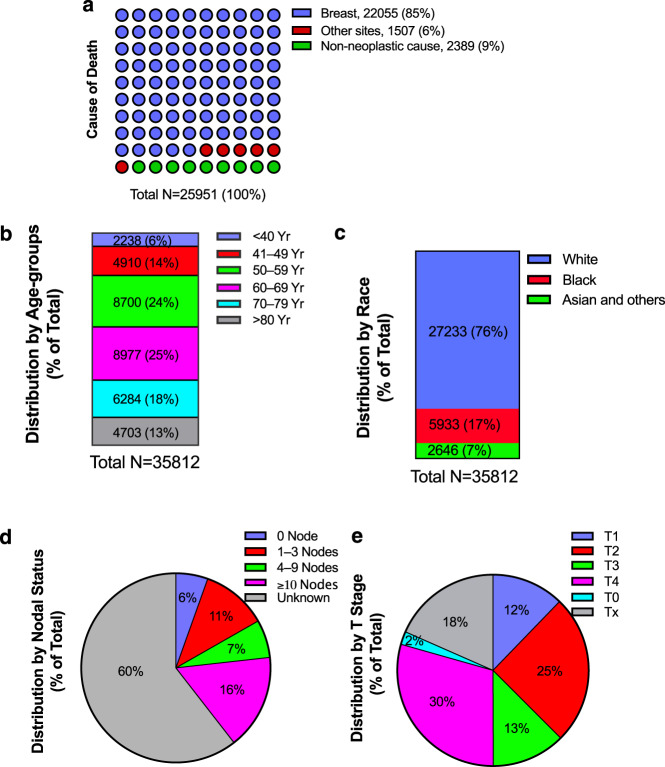
Cause of death and distribution of de novo metastatic breast cancer by clinical factors in the United States. Shown were **a**, cause of death to site in the waffle chart; **b**, and **c**, number, and percentage of patients by age groups and race in the stacked bar chart; and **d** and **e**, distribution of the metastatic population by nodal status and T stages in the pie chart. N, number; Yr, year.

Total 38810 patients (5.4%), with median age of 66 years, had metastatic breast cancer at diagnosis among all breast cancer cases in the SEER database between January 1, 2004, and December 31, 2015. Median follow-up was 72 (range 1 to 155) months; median OS was 28 (range 1 to 155) months in 35812 patients after excluding those diagnosed at autopsy or with death certificates and cases with a survival time of less than one month. There were 25951 death events, in which 22055 (85.0%) were attributable to breast cancer (Fig. 1a). Patient demographics and tumor characteristics of the population were shown in Fig. 1 and Table 1. The prevalence of de novo metastatic breast disease was 25% in 60 to 69 age group, followed by 24% in 50 to 59 age group. Proportions of patients were 76 and 17% in white and black ethnicities, respectively; and there was 1.1% of males in the population. The leading grade was the poorly differentiated (grade 3; 38.3%), followed by moderately differentiated (grade 2; 30.2%) and well differentiated (grade 1; 6.1%). There were 66.5% estrogen receptor-positive tumors and 21.4% HER2-positive tumors in the de novo metastatic population.

**Table 1:** Distribution of the population demographics and clinicopathologic factors^a^.

Variable	Patient *N* (%); total 35812^b^
Gender	
Female	35,404 (98.9)
Male	408 (1.1)
Tumor grade	
Well differentiated	2187 (6.1)
Moderately differentiated	10,813 (30.2)
Poorly differentiated^a^	13,708 (38.3)
Unknown	9104 (25.4)
Histology type	
Unspecified	652 (1.8)
Epithelial	2455 (6.8)
Squamous cell^a^	65 (0.2)
Adenocarcinoma	2415 (6.7)
Adnexal and skin appendage	90 (0.3)
Mucinous	351 (1)
Ductal and lobular	29,579 (82.6)
Complex epithelial^a^	181 (0.5)
Fibroepithelial	24 (0.1)
Estrogen receptor	
Negative^a^	8164 (22.8)
Positive	23,829 (66.5)
Unknown	3819 (10.7)
HER2^a^	
Negative^a^	13,438 (67.6)
Positive	4250 (21.4)
Unknown	2192 (11.0)
Radiation therapy	
No	23,689 (66.1)
Yes	12,123 (33.9)
Chemotherapy	
No	16,652 (46.5)
Yes	19,160 (53.5)
Surgery	
Not performed^a^	23,225 (64.9)
Yes	12,587 (35.1)

^a^ Staged according to the adjusted American Joint Committee on Cancer (AJCC) sixth edition. Squamous cell histology included one basal and two transitional cell and the complex epithelial histology included one case of acinar cell; The poorly differentiated tumors included 400 cases of undifferentiated breast tumors; 47 cases of estrogen receptor borderline tumors were classified as estrogen receptor-negative; HER2 status was not reported to SEER before 2010, thus there were total 19880 cases included for HER2 category, and HER2 borderline (540 cases) was defined as HER2-negative; Surgery not performed included 150 cases of unknowns.

^b^ All patients with a survival time of 1 to 155 months in the SEER database from 2004 to 2015.

*HER2* human epidermal growth factor receptor 2, *N* number.

### Nodal involvement associated with all-cause and breast cancer-specific mortalities

**Fig. 2:**
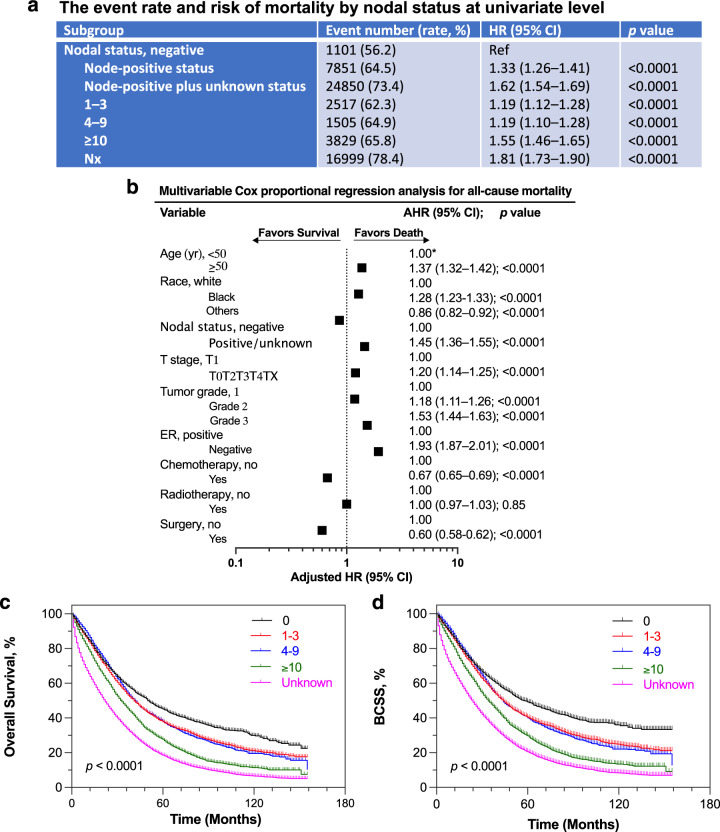
All-cause mortality, and OS and BCSS in patients with metastatic breast cancer by nodal status. **a** Event rate and risk of mortality by nodal status at univariate level. HR and 95% CI of ratio were computed by Cochran-Mantel-Haenszel test. HR, hazard ratio; Ref, reference population. **b** Multivariable Cox model and all covariates in association with the number of death events in the Cox model were analyzed by likelihood ratio test and shown in the forest plot; data were presented as adjusted HR (AHR) with 95% CI. *1.00, reference group. AHR of 1.00 indicates a lack of association; greater than 1.00, an increased risk of mortality; and less than 1.00, a decreased risk of mortality in the forest plot. ER, estrogen receptor. **c** Kaplan–Meier analysis of OS and **d** of BCSS stratified by node-negative, 1–3, 4–9, 10 nodes or greater involvements, and node unknown status.

Compared with node-negative disease, node-positive status (hazard ratio [HR] = 1.33, 95% confidence interval [CI] of ratio 1.26–1.41, *p* < 0.0001) and node-positive plus node-unknown status (HR = 1.62, 95% CI of ratio 1.54–1.69, *p* < 0.0001) were associated with an increased probability of mortality at univariate level (Fig. 2a). The risk of death was incrementally mounted in patients with increasing number of nodes stratified by 1 to 3, 4 to 9, 1.55 in ≥10 node involvements and unknown nodes. We then performed multivariable Cox proportional hazards regression analyses to assess whether the increased probability of mortality in patients with node-positive and node-unknown disease was independent of potential confounding effects of clinicopathologic factors. Positive plus unknown nodes were associated with a 45% increase in the risk of mortality (adjusted HR = 1.45, 95% CI, 1.36–1.55, *p* < 0.0001), controlling for age, race, T stage, grade, estrogen-receptor status and/or HER2 status as well as treatment factors (Fig. 2b; Supplementary Fig. 1). Similar results were obtained in the time-to-event analyses for overall survival (OS) and breast cancer-specific survival (BCSS) using Kaplan–Meier method. The survival proportions at 60 months (5 years) were 45.2% in node-negative, 37.9% in 1 to 3 node involvements, 38.1% in 4 to 9 node involvements, 27.7% in ≥10 node involvements, or 18.6% in node unknown status, respectively (Fig. 2c). The median OS was 51 months in node-negative patients and 27 months in node-positive plus node-unknown metastatic breast cancer patients (Supplementary Fig. 2). Moreover, aggregating lymph node involvements were associated with the increased probabilities of breast cancer-specific mortality (BCSM; Supplementary Fig. 1). BCSS at 5 years (60 months) were 49.2, 40.4, 40.1, 29.7 or 20.5% months in node-negative, 1 to 3, 4 to 9, ≥10 nodes or unknown node status (*p* < 0.0001; Fig. 2d).

### T stage associated with all-cause and breast cancer-specific mortalities

**Fig. 3:**
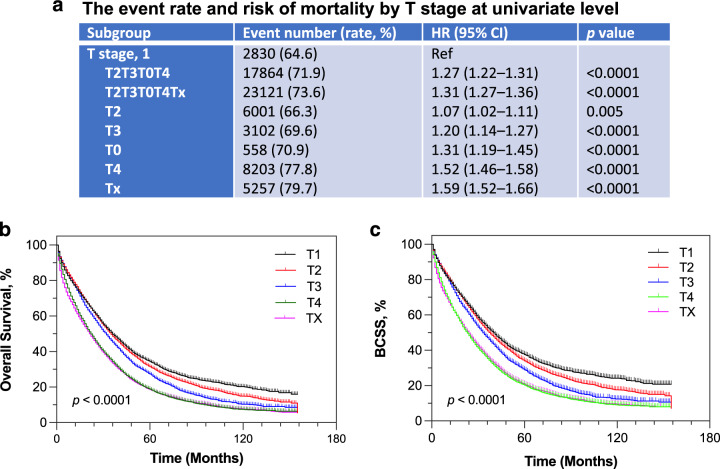
Event rate and all-cause mortality by T stage, and OS and BCSS by T stage in patients with metastatic breast cancer. **a** Event rate and risk of mortality by T stage at univariate level. HR and 95% CI of ratio were computed by Cochran–Mantel–Haenszel test. HR, hazard ratio; Ref, reference population. Kaplan–Meier estimate **b**, for OS and **c**, for BCSS stratified by T1, T2, T3, T4 and Tx. Tick-marks represented the censored patients at the time of last known alive. BCSS, breast cancer-specific survival; OS, overall survival.

We next evaluated another locoregional factor, T stage, in association with the risk of mortality in the de novo metastatic population. As shown in Fig. 3a, there was an increased probability of mortality, compared to T1, in T2T3T4T0Tx stages combined (HR = 1.31; 95% CI of ratio 1.27–1.36; *p* < 0.0001); the probability of death was incrementally escalated in advancing T stages stratified by T2, T3, T0, T4, and Tx. In the multivariable Cox regression model, T stage (adjusted HR = 1.20, 95% CI 1.14–1.25, *p* < 0.0001) was independently associated with the risk of death after adjustment for clinicopathologic factors with and without HER2 (Fig. 2b; Supplementary Fig. 1). The survival proportions were 34.3% in T1, 31.4% in T2, 27.3% in T3, 18.8% in T4 and 18.6% in Tx at 60 months (5 years), respectively (Fig. 3b). Median OS was 27 months in patients with T2, T3, T4, T0 and TX combined, and 36 months in those with T1 (Supplementary Fig. 2). In addition, local tumor stage and its incremental advances were associated with an increased probability of BCSM (Supplementary Fig. 1). Correspondingly, BCSS at 5 years were 37.6, 34.0, 28.9, 20.3 and 20.8% months in T1, T2, T3, T4 and TX, respectively (*p* < 0.0001; Fig. 3c).

### Nodal status and T stage had outcome impact in metastatic breast cancer subtypes

**Fig. 4:**
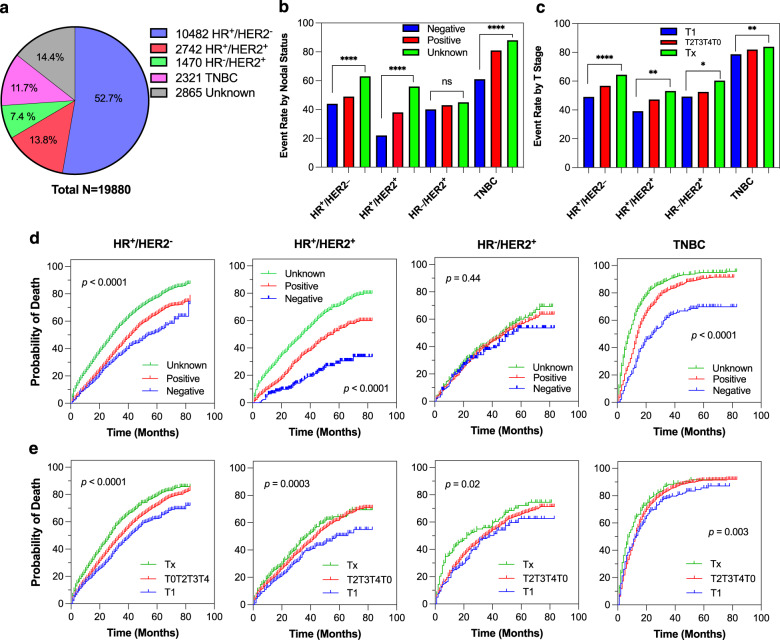
All-cause death by nodal status and T stage in metastatic breast cancer subtypes. **a** Number, and percentage of distribution of breast cancer subtypes in de novo metastatic patients. **b** Event rate by nodal status in HR^+^/HER2^–^, HR^+^/HER2^+^, HR^–^/HER2^+^ and TNBC subtypes. **c** Event rate by T stage in HR^+^/HER2^–^, HR^+^/HER2^+^, HR^–^/HER2^+^ and TNBC subtypes. **d** Kaplan–Meier curves for death stratified by node-negative, node-positive, and node-unknown in the subtypes. **e**, Kaplan-Meier analysis for death stratified by T1, T2T3T4T0 combined, and Tx in the subtypes. Negative, node-negative; Positive, node-positive, TNBC, triple-negative breast cancer. All determined by log-rank test: *p < 0.05, **p < 0.01, ****p < 0.0001, and ns, not significant.

We investigated the effects of nodal involvement and T stage with all-cause mortality in metastatic breast cancer subtypes^18^. The distribution of the metastatic subtypes was 52.7, 13.8, 7.4, or 11.7% in hormone-receptor-positive/HER2-negative (HR^+^/HER2^–^), HR^+^/HER2^+^, HR^–^/HER2^+^ or triple-negative breast cancer (TNBC) (Fig. 4). The time-to-event analyses revealed that mortality curves were significantly different in HR^+^/HER2^–^, HR^+^/HER2^+^ and TNBC subtypes (*p* < 0.0001 each) except HR^–^/HER2^+^ subtype stratified by node-negative, node-positive, and node-unknown status. In particular, a striking difference in mortality rates among these categories were observed in HR^+^/HER2^+^ and TNBC subtypes. In addition, the mortality curves were statistically differed in all four subtypes stratified by T1, T2T3T4T0 combined, and Tx stages (*p* < 0.05 each).

## Discussion

Regional lymph node involvement and local tumor size are established risk factors for nonmetastatic cancer, commonly used for risk assessment^20,21^. In this study, both nodal status and T stage in relation to the risk of mortality were investigated in metastatic breast cancer at diagnosis. With the unbiased population data, our findings demonstrated explicit evidence that regional nodal status and T stage were associated with cumulative all-cause mortality, respectively, independent of known clinicopathologic measurements. They remained statistically significant when HER2 was included in the multivariable Cox regression model despite with the reduced population size (Supplementary Fig. 1a).

Compared with node-negative metastatic disease, probabilities of death were 19, 19, 55 and 81% higher in 1 to 3, 4 to 9, 10 or more node involvements, and node unknown metastatic breast cancers, separately. Nodal status overall and their incremental progresses were also significantly associated with breast cancer-specific mortality. Thus, node-negative patients had much better OS and BCSS than those who had node-positive disease and node-positive plus node-unknown status, with the survival rates more than quadrupled at the end of about 13-year follow-up.

The population-based data also showed that patients with the incrementally advanced T stages had higher risk of mortality than individuals with T1 tumors. As such, these represented 7, 20, 52 and 59% increased probability of death in T2, T3, T4 and TX breast cancers, respectively. It is important to note that the magnitude of mortality risks stratified by nodal status was greater than that stratified by T stage (Figs. 2 and 3). That said, cancer cells spread to the regional lymph nodes were relatively more aggressive than those at local breast site and were thus probably instigated to a higher degree of mortality difference between negative and positive lymph node categories. Together, with tumor advancement in the breast and regional areas, the probability of mortality was significantly increased, independent of other clinicopathologic and treatment factors. The data supports application of TN staging in the de novo metastatic breast disease as in primary malignancies^22,23^. Moreover, we identified both unknown tumor stage and unknown node diseases as the aggressive subsets of de novo metastatic breast cancer and carried higher probabilities of death, consistent with the breast cancer statistics reported by DeSantis and colleagues in CA: a Cancer Statistics for Clinicians^11^.

According to the breast cancer statistics and cohort study, the prevalence of distribution of breast cancer subtypes for primary breast cancer in the United States was 68% in HR^+^/HER2^–^, 10% in HR^+^/HER2^+^, 10% in TNBC, 4% in HR^–^/HER2^+^ or 7% in unknown subtype in early breast cancer^11,24^. As revealed in Fig. 4, the rate of HR^+^/HER2^–^ breast cancer subtype was 52.7% in patients with de novo metastatic breast cancer, which is lower than primary breast cancer, consistent with the breast cancer statistics and other data^11,14^. We demonstrated a proof of impact of the locoregional tumor load on the mortality risk in metastatic breast cancer subtypes. Excluding node-unknowns, death event rates were 44% in node-negative and 49% in node-positive cases in HR^+^/HER2^-^ subtype (*p* = 0.0004); 22 and 61% in node-negative, and 38 and 79% in node-positive HR^+^/HER2^+^, and TNBC subtypes, respectively (*p* < 0.0001 each). According to T stage without Tx, event rates were 49% in T1 and 57% in T2T3T4T0 stages combined in HR^+^/HER2^-^ subtype (*p* < 0.0001); 39% in T1 versus 47% in other T stages in HR^+^/HER2^+^ subtype (*p* = 0.003). Therefore, nodal involvement and T stage were associated with the mortality risk in the metastatic breast cancer subtypes and could have an impact on the management of metastatic breast cancer^25,26^.

In conclusion, the risk of all-cause mortality was significantly higher in patients who presented with distant metastasis at diagnosis and concurrently exhibited regional node involvement and larger breast tumors under the circumstance of controlling for clinicopathologic and treatment factors. This is an unbiased population-based study in the context of the SEER data, with authority, compliance of regulations, and standardization of data registration, recodes and cancer staging. Recognition and integration of locoregional risk into the risk assessment and management could ultimately lead to an improvement of survival landscape in patients with de novo metastatic breast cancer. It may shed light on the recurrent metastatic breast cancer and other cancer types.

## Methods

### De novo metastatic breast cancer population

This study included all patients who were diagnosed with de novo metastatic breast disease in the United States between January 1, 2004, and December 31, 2015, and the dataset also included human epidermal growth factor receptor 2 (HER2) status and breast cancer subtype data since 2010 (Table 1). Derived from the American Joint Committee on Cancer (AJCC) Stage Group 6th edition (2004–2015), nodal status in the de novo metastatic breast cancer included node-negative (no regional lymph node involvement), 1 to 3 lymph node, 4 to 9 lymph nodes, ≥10 lymph involvements that the latter included cases of the documented positive node but the number is unspecified, and node status unknown (nodal status cannot be assessed). T stages in metastatic disease were categorized as T1 (tumor size ≤ 2.0 cm), T2 (>2.0 to ≤5.0 cm), T3 (>5.0 cm) and T4 (tumor of any size with direct extension into the chest wall and/or skin and this includes inflammatory breast cancer) as well as T0 (no evidence of primary tumor in the breast but with distant metastasis) and Tx (primary tumor cannot be assessed). All patients were followed-up until the end of 2016, based on the November 2018 submission. The SEER data are de-identified, and no ethics approval and no written informed consent to participate the study are required from the Office of Human Research Protections, National Institutes of Health (Bethesda, Maryland).

### Clinical endpoints and statistical analysis

The main outcome was the risk of all-cause mortality or OS. It is an estimate from the date of diagnosis to the date of death due to any cause or to the date last known alive^27,28^. BCSM or BCSS was considered as a secondary outcome. It is an estimate from the date of diagnosis to the date of breast cancer-specific death or to the date last known alive. The Cochran-Mantel-Haenszel statistic was employed to evaluate the effect of nodal status and T stage overall and stratified by their incremental advancements with the risk of all-cause mortality and BCSM at univariate level. Kaplan–Meier analysis and log-rank test assessed the association of nodal involvement and T stage with OS and BCSS in the population and BCSM in metastatic breast cancer subtypes. Multivariable Cox proportional hazards regression models estimated independent performance and corresponding 95% CI of T stage and number of regional nodes positive after adjusting for clinicopathologic measurements including age, race, tumor grade, ER status, and treatment factors with or without HER2 status. A likelihood ratio test assessed the performance of all covariates in association with the number of death events in the multivariable Cox models. All statistical tests were two-sided, and a significance level was pre-specified with a *p* value less than 0.05. The data accession and statistical analyses were performed using the SEER*Stat software (version 8.3.8), Prism version 9 (GraphPad software), and Lifelines version 0.26.3 (Python) and/or R version 4.0.2 (R Foundation software).

### Reporting summary

Further information on research design is available in the Nature Research Reporting Summary linked to this article.

## Supplementary information

Supplementary Information

REPORTING SUMMARY

## Data Availability

The access to the SEER 2004–2016 Research Database File can be obtained through SEER*Stat (https://seer.cancer.gov/seerstat/) after data-use agreement approval by the Surveillance, Epidemiology, and End Results Program, National Cancer Institute, Bethesda, Maryland, United States.

## Code availability

Requests for the code used to analyze the above data can be fulfilled by SEER*Stat (https://seer.cancer.gov/seerstat/), and software listed in the Methods.
