# Supplementary material for: Locoregional tumor burden and risk of mortality in metastatic breast cancer
Source: NPJ Precis Oncol. 2022 Apr 5;6:22. doi: 10.1038/s41698-022-00265-9 (PMC8983737; doi:10.1038/s41698-022-00265-9)
Supplement: Supplementary file 1 — Supplementary Information [file 41698_2022_265_MOESM1_ESM.pdf]

Supplementary Figure 1

a **Multivariable Cox proportional regression analysis for all-cause mortality**

Variable	AHR (95% CI); *p* value
Surgery, no	1.00*
Yes	0.56 (0.54–0.59); <0.005
Radiotherapy, no	1.00
Yes	0.97 (0.92–1.01); 0.15
Chemotherapy, no	1.00
Yes	0.64 (0.61–0.67); <0.005
HER2, Negative	1.00
Positive	0.67 (0.63–0.70); <0.005
ER, Negative	1.00
Positive	0.38 (0.36–0.41): <0.005
Tumor grade, 1	1.00
Grade 2/3	1.49 (1.36–1.62); <0.005
Race, Black	1.00
White/others	0.78 (0.73–0.82); <0.005
Age (yr), < 50	1.00
≥ 50	1.39 (1.31–1.46); <0.005
T Stage, T 1	1.00
T0/T2/T3/T4/Tx	1.23 (1.15–1.31); <0.005
Nodal status, 0	1.00
Positive/unknown	1.42 (1.28–1.58); <0.005

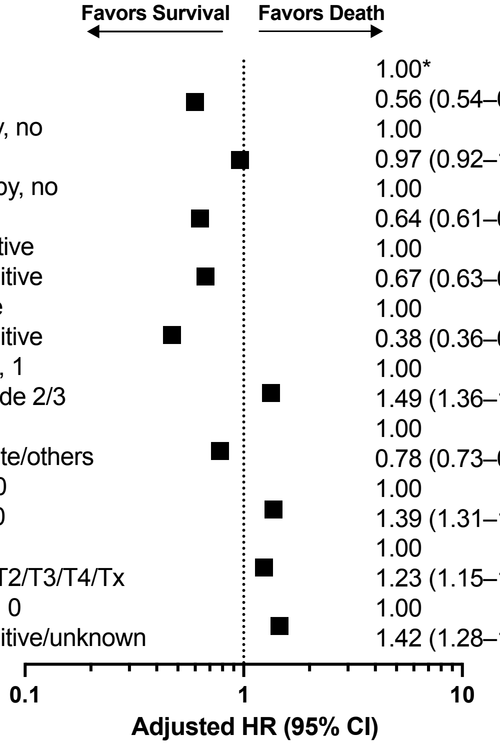

b **The event rate and risk of breast cancer-specific mortality by nodal status and T stage at univariate level**

Subgroup	Event number (rate, %)	HR (95% CI)	*p* value
Nodal status, negative	887 (50.9)	Ref	
Node-positive status	6862 (61.3)	1.39 (1.30–1.48)	<0.0001
Positive plus unknown status	21168 (70.2)	1.66 (1.58–1.75)	<0.0001
1–3	2168 (58.7)	1.24 (1.15–1.34)	<0.0001
4–9	1320 (61.9)	1.26(1.16–1.37)	<0.0001
≥10	3374 (62.9)	1.62 (1.52–1.74)	<0.0001
Nx	14306 (75.4)	1.87 (1.78–1.96)	<0.0001
T stage, 1	2354 (60.3)	Ref	
T2T3T0T4	15459 (68.9)	1.31 (1.26–1.36)	<0.0001
T2T3T0T4Tx	19815 (70.5)	1.35 (1.30–1.40)	<0.0001
T2	5145 (62.8)	1.09 (1.04–1.15)	0.0003
T0	399 (63.5)	1.17 (1.05–1.31)	0.006
T3	2733 (66.8)	1.26 (1.19–1.33)	<0.0001
T4	7182 (75.4)	1.60 (1.53–1.67)	<0.0001
Tx	4356 (76.5)	1.62 (1.54–1.70)	<0.0001

Supplementary Figure 1 All-cause mortality with HER2 by multivariable Cox proportional regression analysis and BCSM by nodal status and T stage. a, Multivariable Cox model with HER2, and all covariates in association with the number of death events in the Cox model for all-cause mortality were analyzed by likelihood ratio test; data are presented as adjusted HR (AHR) with 95% CI. *1.00, reference population. AHR of 1.00 indicates a lack of association; greater than 1.00, an increased risk of mortality; and less than 1.00, a decreased risk of mortality in the forest plot. ER, estrogen receptor; HER2, human epidermal growth factor receptor 2. **b,** Event rate and risk of BCSM by nodal status and T stage at univariate level. HR and 95% CI of ratio were computed by Cochran-Mantel-Haenszel test. BCSM, breast cancer-specific mortality; HR, hazard ratio; Ref, reference population.

Supplementary Figure 2

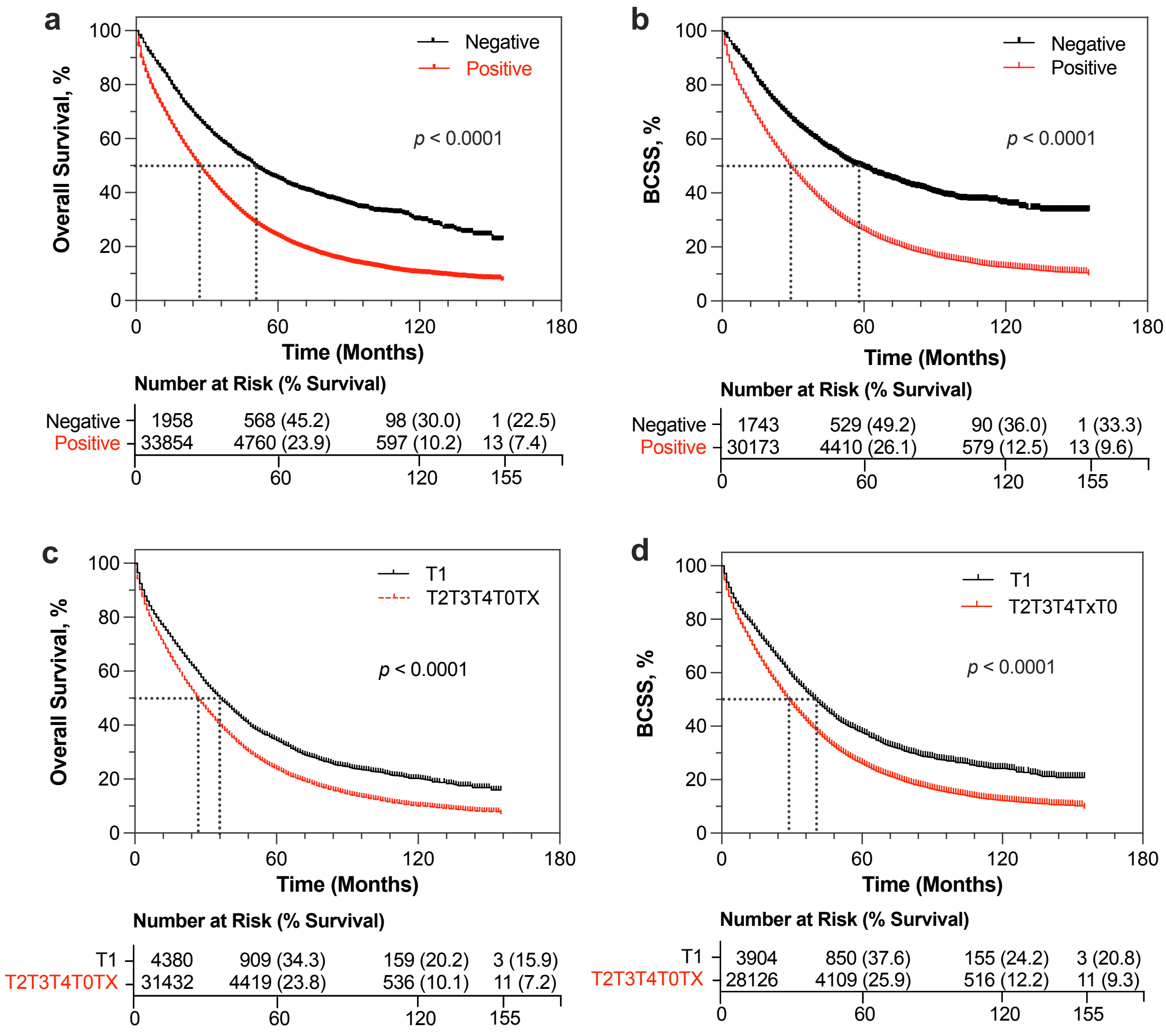

Supplementary Figure 2 Survival of patients with metastatic breast cancer by nodal status and T stage. **a,** Kaplan-Meier analysis of OS and **b,** of BCSS by comparing node-negative to node-positive and unknown. **c,** Kaplan-Meier estimate of OS and **d,** of BCSS by T1 versus T2T3T4T0Tx combined. Tick-marks represented the censored patients at the time of last known alive. BCSS, breast cancer-specific survival; Negative, node-negative; Positive, node-positive; OS, overall survival, Unknown, node status unknown.
