# Supplementary material for: Locoregional tumor burden and risk of mortality in metastatic breast cancer
Source: NPJ Precis Oncol. 2022 Apr 5;6:22. doi: 10.1038/s41698-022-00265-9 (PMC8983737; doi:10.1038/s41698-022-00265-9)
Supplement: Supplementary file 2 — REPORTING SUMMARY [file 41698_2022_265_MOESM2_ESM.pdf]

nature portfolio

Corresponding author(s): Dr Sherry Yang

Last updated by author(s): Feb 14, 2022

Reporting Summary

Nature Portfolio wishes to improve the reproducibility of the work that we publish. This form provides structure for consistency and transparency in reporting. For further information on Nature Portfolio policies, see our Editorial Policies and the Editorial Policy Checklist.

Statistics

For all statistical analyses, confirm that the following items are present in the figure legend, table legend, main text, or Methods section.

n/a	Confirmed	
☐	☒	The exact sample size (n) for each experimental group/condition, given as a discrete number and unit of measurement
☒	☐	A statement on whether measurements were taken from distinct samples or whether the same sample was measured repeatedly
☐	☒	The statistical test(s) used AND whether they are one- or two-sided<br>*Only common tests should be described solely by name; describe more complex techniques in the Methods section.*
☐	☒	A description of all covariates tested
☒	☐	A description of any assumptions or corrections, such as tests of normality and adjustment for multiple comparisons
☐	☒	A full description of the statistical parameters including central tendency (e.g. means) or other basic estimates (e.g. regression coefficient) AND variation (e.g. standard deviation) or associated estimates of uncertainty (e.g. confidence intervals)
☐	☒	For null hypothesis testing, the test statistic (e.g. F, t, r) with confidence intervals, effect sizes, degrees of freedom and P value noted<br>*Give P values as exact values whenever suitable.*
☒	☐	For Bayesian analysis, information on the choice of priors and Markov chain Monte Carlo settings
☒	☐	For hierarchical and complex designs, identification of the appropriate level for tests and full reporting of outcomes
☒	☐	Estimates of effect sizes (e.g. Cohen's d, Pearson's r), indicating how they were calculated

Our web collection on statistics for biologists contains articles on many of the points above.

Software and code

Policy information about availability of computer code

Data collection	The data accession was performed using the SEER*Stat software (version 8.3.8)
Data analysis	The statistical analyses were performed using the SEER*Stat software (version 8.3.8), Prism version 9 (GraphPad software), and Lifelines version 0.26.3 (Python) and/or R version 4.0.2 (R Foundation software).

For manuscripts utilizing custom algorithms or software that are central to the research but not yet described in published literature, software must be made available to editors and reviewers. We strongly encourage code deposition in a community repository (e.g. GitHub). See the Nature Portfolio guidelines for submitting code & software for further information.

Data

Policy information about availability of data

All manuscripts must include a data availability statement. This statement should provide the following information, where applicable:

- Accession codes, unique identifiers, or web links for publicly available datasets
- A description of any restrictions on data availability
- For clinical datasets or third party data, please ensure that the statement adheres to our policy

The population data are available at the SEER Program database, National Cancer Institute, Bethesda, Maryland, United States, and accessed via https://seer.cancer.gov/seerstat/.

Field-specific reporting

Please select the one below that is the best fit for your research. If you are not sure, read the appropriate sections before making your selection.

☒ Life sciences ☐ Behavioural & social sciences ☐ Ecological, evolutionary & environmental sciences

For a reference copy of the document with all sections, see nature.com/documents/nr-reporting-summary-flat.pdf

Life sciences study design

All studies must disclose on these points even when the disclosure is negative.

Sample size	Total 38810 patients (5.4%) had metastatic breast cancer at diagnosis among all breast cancer cases in the SEER database between January 1, 2004, and December 31, 2015, and 35812 patients were included in this study after excluding those diagnosed at autopsy or with death certificates and cases with a survival time of less than one month.
Data exclusions	No data were excluded from the analyses of 35812 patients with de novo metastatic breast cancer.
Replication	NA
Randomization	Multivariable Cox proportional hazards regression models estimated independent performance and corresponding 95% CI of T stage and number of regional nodes positive after adjusting for clinicopathologic and treatment factors including age, race, tumor grade, ER status, and treatment factors with or without HER2 status.
Blinding	The patient data is objectively collected by the National Cancer Institute's SEER program.

Reporting for specific materials, systems and methods

We require information from authors about some types of materials, experimental systems and methods used in many studies. Here, indicate whether each material, system or method listed is relevant to your study. If you are not sure if a list item applies to your research, read the appropriate section before selecting a response.

Materials & experimental systems

n/a	Involved in the study	
☒	☐	Antibodies
☒	☐	Eukaryotic cell lines
☒	☐	Palaeontology and archaeology
☒	☐	Animals and other organisms
☐	☒	Human research participants
☐	☒	Clinical data
☒	☐	Dual use research of concern

Methods

n/a	Involved in the study	
☒	☐	ChIP-seq
☒	☐	Flow cytometry
☒	☐	MRI-based neuroimaging

Human research participants

Policy information about studies involving human research participants

Population characteristics	Total 38810 patients (5.4%), with median age of 66 years, had metastatic breast cancer at diagnosis among all breast cancer cases in the SEER database between January 1, 2004, and December 31, 2015. Median follow-up was 72 (range 1 to 155) months; median OS was 28 (range 1 to 155) months in 35812 patients after excluding those diagnosed at autopsy or with death certificates and cases with a survival time of less than one month.
Recruitment	NA
Ethics oversight	The SEER data are de-identified, and no ethics approval and no written informed consent to participate the study are required from the Office of Human Research Protections, National Institutes of Health, Bethesda, Maryland.

Note that full information on the approval of the study protocol must also be provided in the manuscript.

Clinical data

Policy information about clinical studies

All manuscripts should comply with the ICMJE guidelines for publication of clinical research and a completed CONSORT checklist must be included with all submissions.

Clinical trial registration	NA

Study protocol	NA
Data collection	This study included all patients who were diagnosed with de novo metastatic breast disease in the United States between January 1, 2004, and December 31, 2015, and data were collected by the SEER program.
Outcomes	The main outcome was the risk of all-cause mortality or overall survival (OS). It is an estimate from the date of diagnosis to the date of death due to any cause or to the date last known alive. Breast cancer-specific mortality (BCSM) or breast cancer-specific survival (BCSS) was considered as a secondary outcome. It is an estimate from the date of diagnosis to the date of breast cancer-specific death or to the date last known alive.
